# Improved Cell Line IPEC-J2, Characterized as a Model for Porcine Jejunal Epithelium

**DOI:** 10.1371/journal.pone.0079643

**Published:** 2013-11-15

**Authors:** Silke S. Zakrzewski, Jan F. Richter, Susanne M. Krug, Britta Jebautzke, In-Fah M. Lee, Juliane Rieger, Monika Sachtleben, Angelika Bondzio, Jörg D. Schulzke, Michael Fromm, Dorothee Günzel

**Affiliations:** 1 Institute of Clinical Physiology, Charité – Universitätsmedizin Berlin, Campus Benjamin Franklin, Berlin, Germany; 2 Department of Gastroenterology, Division of Nutritional Medicine, Charité, Universitätsmedizin Berlin, Campus Benjamin Franklin, Berlin, Germany; 3 Institute of Veterinary Anatomy, Freie Universität Berlin, Berlin, Germany; 4 Institute of Veterinary Biochemistry, Freie Universität Berlin, Berlin, Germany; University of Florida, United States of America

## Abstract

Cell lines matching the source epithelium are indispensable for investigating porcine intestinal transport and barrier properties on a subcellular or molecular level and furthermore help to reduce animal usage. The porcine jejunal cell line IPEC-J2 is established as an *in vitro* model for porcine infection studies but exhibits atypically high transepithelial resistances (TER) and only low active transport rates so that the effect of nutritional factors cannot be reliably investigated. This study aimed to properly remodel IPEC-J2 and then to re-characterize these cells regarding epithelial architecture, expression of barrier-relevant tight junction (TJ) proteins, adequate TER and transport function, and reaction to secretagogues. For this, IPEC-J2 monolayers were cultured on permeable supports, either under conventional (fetal bovine serum, FBS) or species-specific (porcine serum, PS) conditions. Porcine jejunal mucosa was analyzed for comparison. Main results were that under PS conditions (IPEC-J2/PS), compared to conventional FBS culture (IPEC-J2/FBS), the cell height increased 6-fold while the cell diameter was reduced by 50%. The apical cell membrane of IPEC-J2/PS exhibited typical microvilli. Most importantly, PS caused a one order of magnitude reduction of TER and of trans- and paracellular resistance, and a 2-fold increase in secretory response to forskolin when compared to FBS condition. TJ ultrastructure and appearance of TJ proteins changed dramatically in IPEC-J2/PS. Most parameters measured under PS conditions were much closer to those of typical pig jejunocytes than ever reported since the cell line’s initial establishment in 1989. In conclusion, IPEC-J2, if cultured under defined species-specific conditions, forms a suitable model for investigating porcine paracellular intestinal barrier function.

## Introduction

In intensive pig farming, a significant fraction of piglets die after weaning, in many cases due to infectious diarrhea [1]. Intense research efforts are therefore made to reduce mortality in animal breeding. For molecular studies on mechanisms and signaling pathways between germ exposure and diarrheal effect, porcine cell cultures are highly desirable. However, these cultures are only suitable if they closely match the properties of pig small intestinal epithelium. Thus, for research on intestinal barrier function, cell models have to meet specific physiological requirements: reflecting epithelial architecture, displaying adequate transepithelial resistance (TER) and transport properties, reacting to secretagogues, and expressing bowel-relevant tight junction (TJ) proteins. If these prerequisites have been achieved, the model system will be potentially suitable for studying effects of e.g. nutritional factors.

Non-transformed continuous epithelial cell lines of only few species and gut sections are available so far, e.g. IEC-6 from rat small intestine [2], IEC-18 from rat ileum [3], IPEC-1 from pig ileum and jejunum [4], IPEC-J2 from pig jejunum [4], and PSI from pig small intestine [5].

In contrast to cultures of rodent cells, a unique side aspect of porcine cell culture models is the potential application for human purposes because the pig gastrointestinal tract physiology is highly comparable to that of humans [6]. It immediately stands out, compared to other commonly used intestinal cell lines (CMT-93, TER: 400 Ω·cm^2^ [7]; HT-29/B6, TER: 500 Ω·cm^2^ [8]) and pig bowel mucosa (R^epi^: 55 Ω·cm^2^, [9]), that all porcine cell lines mentioned above exhibit extraordinarily high TER values (1 to 15 kΩ·cm^2^) when believed to be fully differentiated by the respective author [5], [10]-[12]. TER is a key parameter of epithelial tightness and is determined by para- and by transcellular processes [13]. The paracellular pathway between enterocytes is limited by the TJ which is formed by opposing transmembrane TJ proteins and mediates different degrees of tightness. The TJ is of central interest as it forms a barrier against uptake of putatively immunogenic macromolecules and an excessive passage of water, small ions, and other solutes [14]. The transcellular pathway through enterocytes is defined by tissue-specific channels and carriers, passive diffusion of lipophilic solutes, and complex transcytosis of large molecules. The jejunal layer is a leaky epithelium which is defined by a ratio of para- and transcellular resistances as R^para^/R^trans^<1 [15]. With respect to the observed high TER values it is questionable whether IPEC-1, IPEC-J2, and PSI could serve as appropriate models reflecting porcine small intestinal epithelium, however, they are often employed as such.

Before using an *in vitro* cell culture model as an *in vivo* substitute, it has to be characterized functionally, morphologically, and on a molecular level. So far, most work has been carried out on IPEC-J2. Generated in 1989 by Berschneider, IPEC-J2 were judged as a usable model for research on jejunocyte differentiation and ion transport. This result was based on confluent monolayers of cuboidal to columnar-shaped jejunocytes, the presence of typical cell-cell contacts and marker enzymes, inducible Cl^−^ secretion, and adequate TER (549±39 Ω·cm^2^). In the following 17 years little research was done on IPEC-J2. However, during that interval, IPEC-J2 electrophysiology appears to have changed, as in 2006 Schierack *et al.* re-characterized the cell line and found a strongly increased TER (1.2 to 6.5 kΩ·cm^2^, depending on membrane support material) [16]. Nonetheless, a lot of further studies, mainly on microbe-associated adhesion/invasion, were performed, based on the sustained suitability for this research field because relevant parameters had remained unaltered [16]–[20]. However, IPEC-J2 are also used for virus, nutrition, and toxicity research and when transport function and/or barrier properties are relevant, it has to be guaranteed that trans- and paracellular routes represent the *in vivo* situation as closely as possible.

As conventionally cultured IPEC-J2 monolayers differ considerably from porcine intestine, we aimed to establish a porcine jejunocyte cell culture model which closely matches (electro-)physiological pig jejunal properties and epithelial architecture in order to apply it in swine research on transepithelial transport and paracellular intestinal barrier function.

## Materials and Methods

### Cell culture conditions

The non-transformed cell line IPEC-J2 (intestinal porcine epithelial cells from jejunum) were originally derived from jejunal epithelia of unsuckled piglets as described by Berschneider et al. [4]. Properties of this cell line were characterized by Schierack et al. [16]. IPEC-J2 of our present study were a kind gift of Dr. Peter Schierack (Hochschule Lausitz, Senftenberg, Germany) and were used between passage 65 and 80. Cells were either cultured conventionally with 10% fetal bovine serum (FBS) (e.g. [4], [21]) and 1% penicillin/streptomycin (both PAA, Cölbe, Germany) in Dulbecco’s Modified Eagle Medium (DMEM)/F-12/HAM (Sigma, Steinheim, Germany) or cells were cultured species-specifically with 5% adult pig serum (PS) (Sigma), 1% penicillin/streptomycin, 1% insulin/transferrin/selenium (ITS) (100×; Gibco, Germany), and 5 ng/ml epidermal growth factor (EGF) (Sigma) in DMEM/F-12/HAM. Cultures were split weekly in 25 or 75 cm^2^ culture flasks (Beckton Dickinson, France) using Trypsin/EDTA (1×; Sigma). For experiments, cells were seeded (IPEC-J2/PS: 2×10^5^ cells/cm^2^, IPEC-J2/FBS: 1.8×10^5^ cells/cm^2^) on six-well plates (Nunc, Germany) or on membrane supports (Millicell^®^-HA culture plate inserts, area: 0.6 cm^2^, pore size: 0.45 µm; Millipore, Ireland). Three to five supports in each petri dish were cultured for 2 to 3 weeks until TER values were stable and monolayers were analyzed. When cells were grown in six-well plates, growing time necessary to obtain stable TER values was determined from parallel cultures on membrane supports. Cells were grown at 37 °C, 5% CO_2_, and 95% relative humidity. They were fed every other day.

In addition, the following culture media were tested: DMEM/F-12/HAM containing 1% penicillin/streptomycin and 10% PS and DMEM/F-12/HAM containing 1% penicillin/streptomycin, 1% ITS, and 5 ng/ml EGF, supplemented with either 5% FBS, adult goat serum (GS) or adult bovine serum (ABS) (all PAA).

### Ethic statement

Experiments were conducted on intestinal tissue of weaned piglets in strict accordance with the German law for the care and use of experimental animals. All procedures involving animal handling were approved by the local state office of occupational health and technical safety (Landesamt für Gesundheit und Soziales Berlin, Permit Number: G 0347/09).

### Jejunal tissue preparation

Piglets were housed and fed control diets. At age 54±1 days, piglets were sedated with 20 mg/kg BW of ketamine hydrochloride (Ursotamin, Serumwerk Bernburg) and 2 mg/kg BW of azaperone (Stresnil, Jansen-Cilag) and killed by intracardial injection of 10 mg/kg BW of tetracaine hydrochloride, mebezonium iodide, and embutramide (T61, Intervet) and exsanguination (see also [22]). The mid jejunum was removed, cut open, and rinsed with and transported in cooled saline solution (0.9% NaCl, 1 mM CaCl_2_). Jejunal tissue was stripped off the muscle layer and explants either were mounted in Ussing chambers, fixed in 2% paraformaldehyde (PFA) or frozen in liquid nitrogen.

### Solutions and reagents

Standard Ringer solution was used (i) plain (113.6 mM NaCl, 5.4 mM KCl, 1.2 mM MgCl_2_, 1.2 mM CaCl_2_, 21 mM NaHCO_3_, 0.6 mM NaH_2_PO_4_, 2.4 mM Na_2_HPO_4_, 10 mM D(+)-glucose; pH 7.4 when equilibrated with carbogen), (ii) glucose-free, (iii) phosphate-free, (iv) bicarbonate-free, and (v) supplemented with substrates and antibiotics (2.5 mM glutamine, 10 mM D(+)-mannose, 0.5 mM β-OH-butyrate, 50 mg/l piperacillin, 4 mg/l imipenem).

In order to block apical Na^+^ and K^+^ channels in IPEC-J2, amiloride (Sigma, final concentration 10 µM), tetraethylammonium (Sigma, 5 mM), and barium chloride (Merck, Darmstadt, Germany, 5 mM) were added apically prior to basolateral administration of forskolin (Calbiochem, Merck, 10 µM) or apical addition of phlorizin (Sigma, 0.5 mM) in plain Ringer bath solution. Apical administration of glucose (Roth, Karlsruhe, Germany, 10 mM) followed cation channel block in glucose-free standard solution. In jejunal tissue studies forskolin, phlorizin, and glucose were applied without previous cation channel block in standard Ringer solution supplemented with substrates and antibiotics or glucose-free standard solution, respectively.

### Electrophysiological measurements

TER progression of IPEC-J2 monolayers was monitored over weeks using a chopstick electrode with automatic height control [23]. TER measurements were corrected for membrane and culture medium resistance and multiplied by the effective monolayer area.

For Ussing chamber experiments, IPEC-J2 monolayers (0.6 cm^2^ effective area) grown on membrane supports were directly mounted [24], whereas stripped jejunal explants were glued to Plexiglas™ rings (0.28 cm^2^ effective area) before being mounted [25]. Ussing chambers and water-jacketed gas lifts were kept at 37°C. Preparations were allowed to equilibrate for 30 (monolayer) or 45 min (tissue), respectively. Resistance of bath solutions alone and electrode offsets were recorded prior to each experiment and subtracted from experimental data. Data produced via chopsticks and in the Ussing chamber were fully compatible.

In order to determine Na^+^/Cl^−^ permeability ratios of IPEC-J2 and jejunal tissue, dilution potential measurements were conducted in the Ussing chamber. After equilibrating monolayers/tissue in phosphate-free Ringer solution, dilution potentials were evoked by iso-osmotically partially replacing NaCl with mannitol. Permeability ratios to Na^+^/Cl^−^ were calculated using the Goldman-Hodgkin-Katz equation as reported before [26]. Dilution potential measurements were performed at 37 °C, except for IPEC-J2/FBS, where part of the experiments were carried out at 25 °C in order to minimize interfering potentials from active, transcellular transport. Nevertheless, values of dilution potentials at both temperatures were not significantly different.

Permeability to fluorescein was determined in the Ussing chamber under voltage clamp conditions. After equilibrating cell layers in standard Ringer solution, fluorescein (Sigma) was added apically (final concentration 100 µM). After 0, 10, 20, 30, 40, and 50 min post administration basolateral samples were taken and replaced with Ringer solution. Fluorescein concentrations were determined at 525 nm fluorescence emission (Infinite M200, Tecan, Crailsheim, Germany) and permeabilities were calculated.

### Impedance spectroscopy

In order to discriminate between epithelial (R^epi^) and subepithelial (R^sub^) resistances, one-path impedance spectroscopy was performed as described previously [25]. Briefly, membrane supports or jejunal explants were mounted in Ussing chambers modified for impedance measurements [27]. A total of 48 frequencies of alternating current (1.3 Hz to 65 kHz) were applied and resulting voltages analyzed through a programmable frequency generator/response analyzer in combination with an electrochemical interface (1250 and 1286, Solartron, Schlumberger, Farnborough, UK). Complex impedance values were plotted as Nyquist diagrams and fitted by circular arcs using least square analysis. The arc intercept with the x-axis at low frequency represents TER and that at high frequencies equals R^sub^. TER minus R^sub^ represents the true epithelial resistance, R^epi^. The frequency at which the semicircle has its minimum is used to calculate the epithelial capacitance (C^epi^) [8]. For splitting R^epi^ in trans (R^trans^)- and paracellular (R^para^) resistances, two-path impedance spectroscopy was conducted as described by Krug *et al.* [8]. In brief, this technique combines one-path impedance spectroscopy and flux measurements of a paracellular marker (i.e. fluorescein) during a Ca^2+^ switch experiment.

### Live cell imaging

Membrane supports covered with IPEC-J2 monolayers were cut out, positioned on a cover slip (apical side down), and covered by a small volume of bicarbonate-free Ringer solution. 20 µl of 4 kDa FITC-dextran (TdB, Upsala, Sweden, 25 mM) were added from the basolateral (upper) side to let the dye flood the paracellular space and surroundings. An inverted confocal laser-scanning microscope (LSM 510 Meta, Zeiss, Jena, Germany) was used to take images in xy and xz plane, which were processed using fiji imaging software [28]. Cell heights of three independent seedings were measured applying Zeiss LSM 510 META software.

### Transmission electron microscopy (TEM)

IPEC-J2 monolayers grown to confluence on membrane supports were stored in Karnovsky’s fixative, washed with sodium cacodylate buffer (0.1 M sodium cacodylate, Roth; adjusted to pH 7.4 using HCl), fixed in osmium tetroxide (1% in 0.2 M sodium cacodylate buffer (pH 7.4), ChemPur, Germany), and washed again. Afterwards, samples were dehydrated (increasing ethanol series, propylene oxide (VWR, Darmstadt, Germany)) and resin-embedded (12.4% Agar 100 resin (w/v), 7.3% dodecenyl succinic anhydride (v/v), 3.6% methylnadic anhydride (v/v), 0.6% benzyldimethylamine (v/v), all Agar Scientific, Essex, UK). The resin was allowed to polymerize at 45 and 60 °C for 24 h each. Ultrathin (70 nm) sections were cut (UltraCut S, Leica, Wetzlar, Germany) and contrasted with uranyl acetate (Serva, Heidelberg, Germany) and lead citrate (Laurylab, Saint-Fons Cedex, France). Images were taken using a transmission electron microscope (EM 902 A, Zeiss). The length of well oriented microvilli and the number of microvilli along >10 µm apical cell membrane of IPEC-J2 in total were obtained from 3 different cells analySIS® software (Version 3.0, Münster, Germany).

### Freeze-fracture electron microscopy (FFEM)

Confluent IPEC-J2 monolayers (grown on 6-well plates) and stripped jejunal tissue were fixed by incubation in 2.5 and 4% phosphate-buffered glutaraldehyde (25% stock solution in water, Serva). Subsequently, preparations were incubated in glycerol (10 and 30%) and finally frozen in liquid nitrogen-cooled Freon 22. Freeze-fracture electron microscopy and morphometric analysis were performed as described before [29].

### Cryosections

PFA-fixed jejunal tissue was dehydrated (stepwise, 10, 20, and 30% sucrose), frozen in liquid nitrogen-cooled methylbutane, and embedded in TissueTek (Sakura, Alphen aan den Rijn, Netherlands). Jejunal tissue directly frozen in liquid nitrogen was immediately embedded in TissueTek. Samples were cut (CM 1900, Leica) in 5 µm thin sections and positioned on glass slides.

### Immunofluorescence staining (IF)

**Cell culture.** IPEC-J2 grown on membrane supports were washed with phosphate-buffered saline containing calcium and magnesium chloride (PBS^+^; Sigma), fixed in methanol at –20 °C for 10 min (or fixed in 2% PFA for 20 min at room temperature (RT) for staining with phalloidin), and washed again. Membranes were stamped out of the plastic support and subsequently incubated with primary antibodies in 0.5% Triton X-100 in PBS^+^ for 2 h at RT, washed, and incubated with secondary antibodies (**Table S1**), 4’,6-diamidino-2-phenylindole dihydrochloride (DAPI, 1 µg/ml; Roche, Grenzach-Wyhlen, Germany), and optionally with Phalloidin-Atto 647N (1:200, Sigma) in 0.5% Triton X-100 in PBS^+^ for 2 h at RT. Membranes were washed with PBS^+^ followed by doubly distilled water and mounted using ProTaqs Mount Fluor (Biocyc, Luckenwalde, Germany).

**Jejunal tissue.** Slices of PFA-fixed tissue were boiled in ethylenediaminetetraacetic acid buffer (1 mM EDTA, pH 8.0 adjusted with NaOH; Merck) for 15 min and then washed in PBS^+^, whereas slices from tissue directly frozen in liquid nitrogen were fixed in methanol at –20°C for 10 min and subsequently washed in PBS^+^. Tissue slices were then permeabilized (5 min, 0.5% Triton X-100 in PBS^+^, RT) and blocked in 5% goat (PAA) or donkey serum (Sigma) plus 1% bovine serum albumin (Roth) for 1 h. Tissue sections were incubated with primary antibodies in blocking solution for 1.5 h at RT, washed, and incubated with secondary antibodies for 1.5 h at RT (**Table S1**). After washing with PBS^+^ and doubly distilled water, slices were embedded using ProTaqs Mount Fluor.

Images were taken with a confocal laser-scanning microscope and software as introduced above. A defined image area (≥400 µm^2^) of IPEC-J2 grown on three independent membrane supports was used to determine cell width assuming an ideally hexagonally shaped cell corpus. Pig jejunal epithelium morphometry was assessed correspondingly using IF staining images.

### Paraffin sections, PAS staining, Morphometry

IPEC-J2 monolayers were fixed in 4% formalin, embedded in paraffin, and sliced transversely in 5 µm thin sections. Slices were deparaffinized and rehydrated (xylene, decreasing ethanol series) prior to periodic acid-Schiff (PAS) reaction. After PAS staining, IPEC-J2 sections were mounted using Corbit-Balsam (I. Hecht, Germany). Images were taken using a conventional fluorescence microscope (BX 60, Olympus, Hamburg, Germany).

To determine the epithelial enlargement factor provided by the villus and crypt surface with reference to the lamina muscularis mucosae, jejunal tissue of 13 piglets was fixed in triplicate for 26 h in Zamboni’s fixation solution, dehydrated in graded series of ethanol, embedded in paraffin, cut to 5 µm thin sections, deparaffinized, and rehydrated. Three slides were prepared for each sample and stained according to H&E standard staining protocols [30]. Five section areas, where at least four villi were cut completely from top to bottom and crypts were cut vertically were analyzed at 50-fold magnification. Enlargement factors were measured according to Wiese *et al.* [31] using the image analysis program NIS-Elements (Nikon, Düsseldorf, Germany).

### Western blot (WB) analyses

WB analyses were performed using standard techniques on IPEC-J2 of four consecutive passages or on stripped jejunal explants of four animals. Primary and secondary antibodies are given in **Table S1**.

The Lumi-Light^PLUS^ Western Blotting Kit (Roche) was used to detect relevant protein bands via the Fusion FX 7 image acquisition system (Vilber Lourmat, Eberhardzell, Germany). Densitometric signal analysis was performed using AIDA software (Raytest, Berlin, Germany).

### Polymerase chain reaction (PCR)

mRNA of IPEC-J2 grown in culture flasks and stripped pig jejunal tissue was extracted using peqGOLD RNAPure™ (peQlab, Erlangen, Germany) and purified using the NucleoSpin^®^ RNA/Protein kit (Macherey-Nagel, Düren, Germany). Reverse transcription was performed via High Capacity cDNA Reverse Transcription (Applied Biosystems, Warrington, UK), and PCR mixtures were prepared using the HotStarTaq^®^ DNA Polymerase (Qiagen, Hilden, Germany). The primer pairs and the anticipated PCR product sizes were: claudin-2 (for: GTTGCCATGCTGCTCCCCAGCTG, rev: TCACACATACCCCGTCAGGCTGTAG; 626 bp), claudin-12 (for: ATGGGCTGTCGGGATGTCCACGCA, rev: TTAGGTGGTGTGGGAAACTACTGG; 734 bp), claudin-15 (for: CACGGGAACGTCATCACCACCA, rev: TCCAGGCCCCCAATGTTGGTGC; 223 bp). DNA amplification was performed thermo cyclically providing gene-specific thermal profiles. Gel pictures were captured using the Luminescent Image Analyzer LAS-1000 (FujiFilm, Düsseldorf, Germany). Signals were verified by sequencing.

### Proliferation assay

Cell proliferation was measured using the Cell Proliferation Reagent WST-1 (Roche) by quantifying the number of metabolically active cells.

IPEC-J2 of four different passages were seeded at a density of 10^4^ cells/well in 96-well microplates and maintained in a cell incubator for either 4 or 72 h. After each growing period, WST-1 Reagent was added and cells were incubated for 1 hour before the absorbance was measured at 450/630 nm using an ELISA reader (Bio-Rad, Munich, Germany). The absorbance at time point 4 h was set as 100%.

### Statistical analysis

Data are expressed as means ± standard error of the mean (SEM). Statistical analyses were carried out using either a two-tailed, unpaired Student’s t-test or a one-way ANOVA with Tukey HSD post hoc test (SPSS, version 20, Chicago, Illinois). Significances are depicted as: *, p<0.05; **, p<0.01; ***, p<0.001.

## Results

### PS culture reduces transepithelial resistance

**Figure 1:**
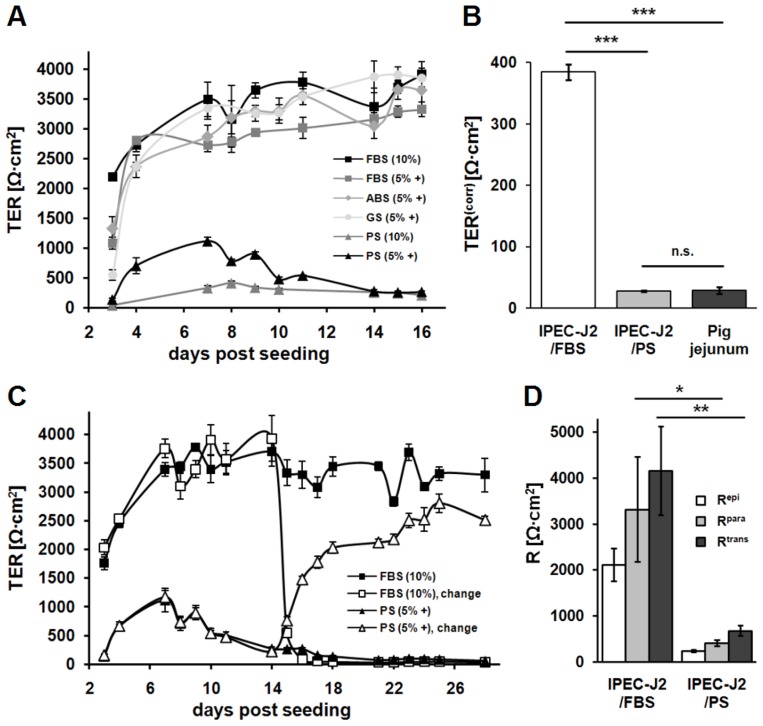
PS reduces transepithelial, transcellular, and paracellular resistance of IPEC-J2. (**A**) Time course of transepithelial resistance (TER) of IPEC-J2, which were cultured using different culture media (FBS, fetal bovine serum; ABS, adult bovine serum; GS, adult goat serum; PS, adult porcine serum; ‘+’ indicates supplementation with ITS and EGF) as indicated in the key. PS prevented cells from developing extremely high TER values over a time period of at least three weeks. Note the TER time course of PS (5% +), which developed a maximum around day 7 and reached a lower plateau level at day 14 post seeding. (n = 4–5 each) (**B**) TER values of IPEC-J2/FBS (white bar) and IPEC-J2/PS (light grey bar) were corrected for surface enlargement by villi and crypts of porcine jejunum (factor 10) (TER^corr^) for comparison with pig jejunal values (dark grey bar). IPEC-J2/FBS exhibited higher TER^corr^ values (n = 20) than pig jejunum (n = 15; ***, p<0.001), whereas TER^corr^ of IPEC-J2/PS (n = 20) was not significantly (n.s.) different from porcine values. (**C**) TER time courses of IPEC-J2/FBS and IPEC-J2/PS were monitored two weeks before and after culture conditions were exchanged as indicated by ‘change’. IPEC-J2 immediately started to develop respective serum-typical TER values. (n = 4–5 each) (**D**) Two-path impedance spectroscopy was employed to determine R^para^ (light grey bars) and R^trans^ (dark grey bars) of IPEC-J2/FBS and IPEC-J2/PS. Significant reduction of both, R^para^ (*, p<0.05, n = 6) as well as R^trans^ (**, p<0.01, n = 6) occurred in PS when compared to FBS culture. R^epi^ (white bars) is calculated as R^para^·R^trans^/R^para^+R^trans^.

IPEC-J2 cultured in medium supplemented with FBS (IPEC-J2/FBS) and grown on permeable membrane supports exhibit huge TER values in the range of 1–15 kΩ·cm^2^ either in a peak (e.g. [19]) or as plateau value (e.g. [32]; **Fig.** **1A**, black and grey squares). In order to compare TER values of IPEC-J2 with values from pig jejunal epithelia, the jejunal surface enlargement by villi and crypts has to be taken into account. Morphometric studies revealed a surface enlargement factor (11.3±0.5, n = 13; range 8.7 to 14.7) which was set 10 for practicability reasons. Having corrected cell culture values (TER^corr^) for the effective jejunal epithelial surface area, values measured *in vitro* and *ex vivo* are directly comparable. As shown in **Fig.** **1B**, TER^corr^ of IPEC-J2/FBS (384±12 Ω·cm^2^, n = 20) still differs by one order of magnitude from TER of pig jejunal epithelium (28±5 Ω·cm^2^, n = 15).

While improving the cell culture protocol to obtain more physiological cell characteristics, fetal bovine serum was replaced by serum from adult pigs. As a striking result, 10% PS instead of FBS prevented cells from developing extreme TER over a time period of at least three weeks (**Fig.** **1A**, grey triangles, black squares). However, culturing IPEC-J2 with culture medium containing 5% PS, ITS, and EGF (**Fig.** **1A**, black triangles) resulted in a more regular cell layer (data not shown). Notably, under PS condition TER developed a maximum around day 7 post seeding and reached a lower plateau level (200–400 Ω·cm^2^) at around day 14 post seeding which was maintained for several days.

To determine whether the effect of lower TER was serum species-specific, brought about by age of the blood donor animal or was caused by medium supplementation with ITS and EGF, appropriate medium compositions were tested (**Fig. 1A**). Neither medium supplemented with 5% FBS, ITS and EGF nor the use of adult bovine or adult goat serum considerably affected TER values and course compared to the 10% FBS condition. In addition, the use of porcine serum obtained from another company (Biochrom, Berlin, Germany) corroborated the effect of lower TER (data not shown). Resulting from this, 10% FBS and 5% PS with ITS and EGF (conventional and species-specific condition, respectively) were chosen for further comparative experiments. Pig jejunal TER (28±5 Ω·cm^2^, n = 15, p<0.001) and TER^corr^ of IPEC-J2/PS (27±1 Ω·cm^2^, n = 20) did not differ significantly (**Fig.** **1B**). When FBS condition was replaced by PS condition (and vice versa) two weeks post seeding, IPEC-J2 immediately started to develop respective serum-typical TER values (**Fig.** **1C**), whereas switches induced by PS were much faster than those induced by FBS.

### IPEC-J2 form a leaky epithelium

R^epi^ (2111±356 Ω•cm^2^, n = 6) of IPEC-J2/FBS is composed of R^para^ (3323±1145 Ω•cm^2^, n = 6) and R^trans^ (4168±967 Ω•cm^2^, n = 6). Similarly but at much lower levels, R^epi^ (242±28 Ω•cm^2^, n = 6) of IPEC-J2/PS consists of R^para^ (411±62 Ω•cm^2^, n = 6) and R^trans^ (679±113 Ω•cm^2^, n = 6) (**Fig.** **1D**). In IPEC-J2 of both kind the ratio R^para^/R^trans^ is below 1 (IPEC-J2/FBS, 0.85±0.2, n = 6; IPEC-J2/PS, 0.69±0.1, n = 6; see also [33]). Despite the high absolute resistances, R^para^/R^trans^<1 indicates a "leaky" epithelium [15] characteristic for the jejunal lining.

### Proliferation rate is not affected by PS

Cell proliferation was not statistically different between IPEC-J2/PS (172±12%, n = 4) and IPEC-J2/FBS (164±9%, n = 4) (**Fig.** **S1**).

### PS improves transport properties

**Figure 2:**
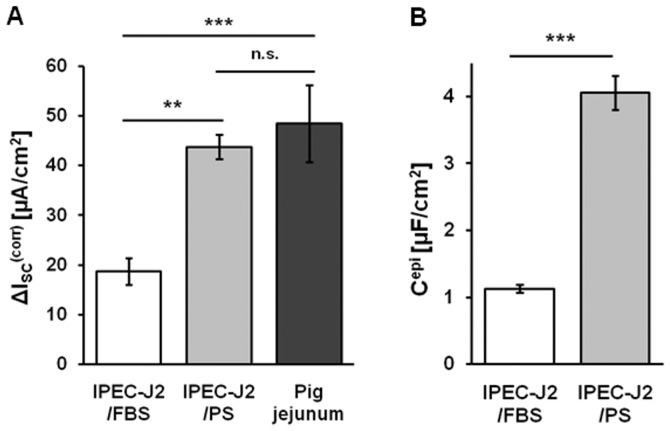
PS elevates active ion transport and membrane capacitance of IPEC-J2. (**A**) Forskolin-induced short-circuit current (▵I_SC_) of IPEC-J2/FBS (white bar) and IPEC-J2/PS (light grey bar) were corrected for surface enlargement by villi and crypts of porcine jejunum (factor 10) (▵I_SC_^corr^) for comparison with pig jejunal values (dark grey bar). Stimulation by forskolin resulted in an increased chloride secretory response in IPEC-J2/PS (n = 11; **, p<0.01) compared to IPEC-J2/FBS (n = 10), which almost reached porcine values (n = 9; n.s.). (**B**) Epithelial capacitance (C^epi^) of IPEC-J2/FBS (white bar) and IPEC-J2/PS (light grey bar) was determined via impedance spectroscopy. In IPEC-J2/PS, C^epi^ was increased (n = 15; ***, p<0.001) compared to IPEC-J2/FBS (n = 22).

Typical features of jejunum in general are ion transport induced by secretagogues as well as glucose absorption via sodium-glucose transporter 1 (SGLT1). IPEC-J2/PS had a significantly stronger secretory response to forskolin (▵I_SC_^corr^, 44±2 µA/cm^2^, n = 11, p<0.01; cell culture values corrected for jejunal surface enlargement) than IPEC-J2/FBS (19±3 µA/cm^2^, n = 10) but comparable to that of porcine tissue (48±8 µA/cm^2^, n = 9) (**Fig.** **2A**). Glucose absorption was either evaluated directly as glucose-stimulated ▵I_SC_ or indirectly as ▵I_SC_ observed upon SGLT1 inhibition by phlorizin. However, both alternatives did not demonstrate any change in glucose-dependent I_SC_ between PS and FBS condition (data not shown).

### PS alters cell morphometry and substructure

**Figure 3:**
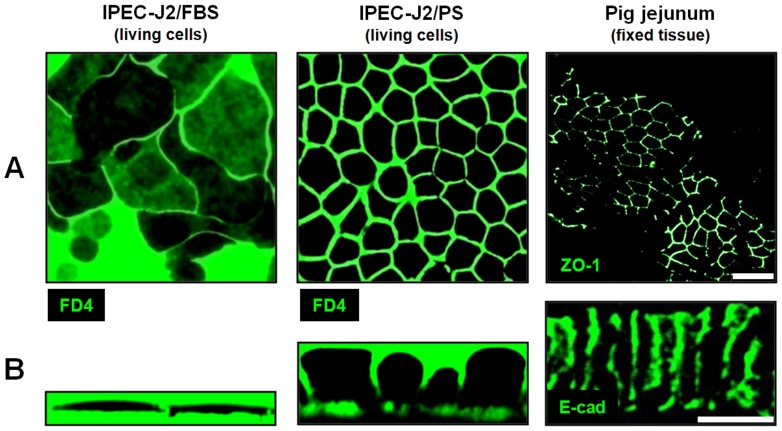
PS optimizes cell dimension of IPEC-J2. (**A**) Horizontal and (**B**) vertical aspects of IPEC-J2 were visualized by live cell imaging using FITC-dextran 4000 (FD4), those of pig jejunocytes by immunofluorescence staining of ZO-1 (**A**) and of E-cadherin (E-cad, **B**). Scale bar: 20 µm.

TER values depend on TJ composition as well as TJ length and cell membrane area per area of the membrane support, whereas I_SC_ depends on the latter only. Total membrane area follows from cell morphology and is reflected by epithelial capacity (C^epi^). C^epi^ was increased by a factor of four in IPEC-J2/PS (4.06±0.25 µF/cm^2^, n = 15, p<0.001), compared to IPEC-J2/FBS (1.12±0.06 µF/cm^2^, n = 22) (**Fig.** **2B**), and thus reached values similar to cylindrical HT-29/B6 cells (3.5-4.5 µF/cm^2^) [34]. Changes in cell morphology were confirmed by live cell imaging. PS caused IPEC-J2 to be smaller in diameter (21.8±1.1 µm, n = 3 independent seedings, analysis of ≥400 µm^2^ each; p<0.01) (**Fig.** **3A**) and taller (17.4±2.2 µm, n = 3 independent seedings, analysis of ≥6 cells each; p<0.001) (**Fig.** **3B**) compared to IPEC-J2/FBS (diameter: 42.2±3.3 µm, height: 3.0±0.3 µm; n = 3). Consequently, the TJ length per unit membrane support doubled when IPEC-J2 were PS- instead of FBS-cultured. IPEC-J2/PS values approximate those of pig jejunocytes (diameter: 8.2±1.0 µm, p<0.01; height: 29.2±0.8 µm; p<0.01; n = 3 independent stainings).

To gain further insight into these morphological changes, the influence of both culture conditions was analyzed ultrastructurally. IPEC-J2/PS exhibited a dome-shaped apical membrane with numerous and long microvilli compared to IPEC-J2/FBS (density, 7.0±1.1 vs. 2.7±0.4 microvilli/µm, n = 3, p<0.05; length, 450±30 nm, vs. 110±10 nm, n = 3, p<0.01; n, number of cells, analysis of >2 µm apical cell membrane each), (**Figs.** **S2A**, **B**). Tight junctional structures were located at the apical-most part of cell-cell contacts, but due to the dome shape of the apical cell membrane, IPEC-J2/PS TJs did not represent the maximum cell height (**Figs.** **S2A**, **B**). The amorphous substance (**Fig.** **S2A**, labeled with M) which is mostly present in IPEC-J2/PS could be identified as neutral mucopolysaccharides (**Fig.** **S2C**).

### PS optimizes tight junction ultrastructure

**Figure 4:**
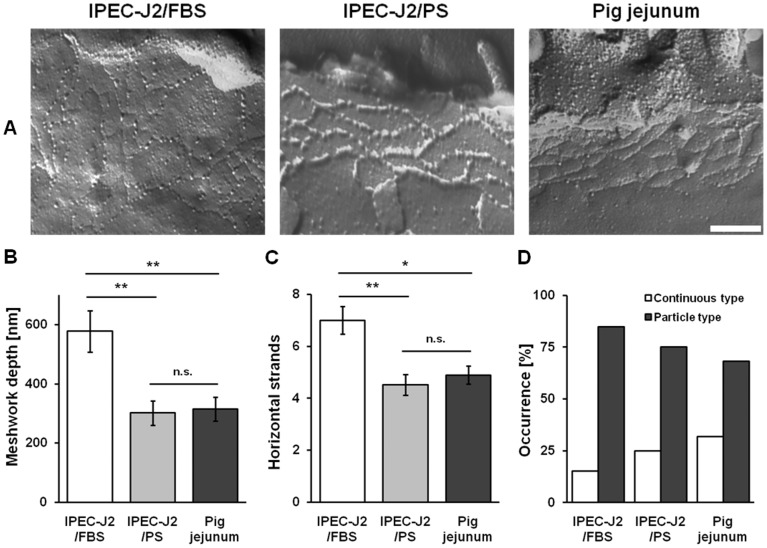
PS approximates tight junction ultrastructure of IPEC-J2 to pig jejunum. (**A**) Freeze-fracture images of IPEC-J2/FBS, IPEC-J2/PS, and pig jejunal tissue (scale bar: 200 nm). (**B**) Morphometric analysis of the TJ meshwork depth, (**C**) the number of horizontal strands, and (**D**) the TJ strand type (continuous vs. particle type). IPEC-J2/PS, n = 22; IPEC-J2/FBS, n = 23; pig jejunum, n = 20; n.s., not significant; *, p<0.05; **, p<0.01.

Detailed analysis was performed for TJ ultrastructure. Neither the TJ meshwork depth nor the number of horizontal strands were significantly different between IPEC-J2/PS (meshwork depth, 302±41 nm; number of horizontal strands, 4.52±0.4; n = 22) and porcine jejunocytes (meshwork depth, 315±41 nm; number of horizontal strands, 4.88±0.35; n = 23) (**Figs.** **4A, B, C**). In contrast, IPEC-J2/FBS differed considerably (meshwork depth, 577±70 nm, p<0.01; horizontal strands, 7.00±0.53, p<0.05; both n = 20) from jejunal tissue. The occurrence of continuous type TJ strands was lowest in IPEC-J2/FBS, medium in IPEC-J2/PS and high in pig jejunal epithelium, whereas an inverse incidence of particle type structures was observed (**Fig. 4D**). Thus, there was no correlation between the presence of continuous TJ strands and high TER values.

### PS increases epithelial permeability

**Figure 5:**
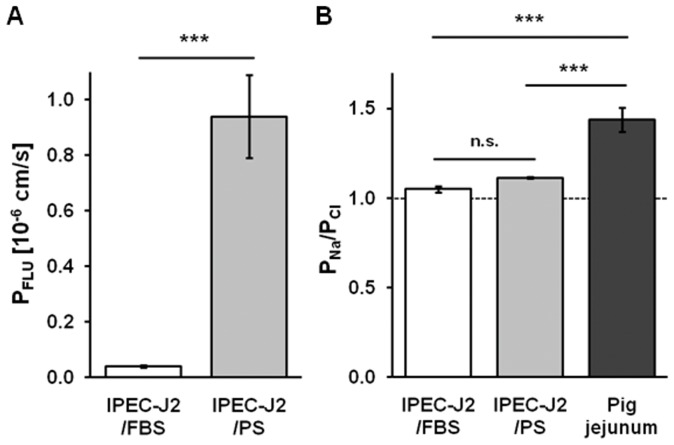
PS affects permeability to fluorescein but not charge selectivity of IPEC-J2. (**A**) Paracellular permeability to fluorescein (P_FLU_) of IPEC-J2/FBS (n = 7) and IPEC-J2/PS (n = 8). (**B**) Permeability ratio for sodium and chloride (P_Na_/P_Cl_), indicating paracellular charge selectivity, as determined by dilution potentials measurements. The broken line indicates no charge selectivity. Pig jejunal tissue (n = 12), IPEC-J2/PS (n = 13), IPEC-J2/FBS (n = 11); n.s., not significant; ***, p<0.001.

TJ composition implies structural aspects and is therefore crucial for paracellular barrier properties including permeability to fluorescein and inorganic ions. The permeability to fluorescein of IPEC-J2/PS (0.94±0.15 10^−6 ^cm/s, n = 7, p<0.001) was dramatically higher than of IPEC-J2/FBS (0.04±0.004 10^−6 ^cm/s, n = 8) and thus reached values similar to HT-29/B6 cells (0.86±0.14 10^−6 ^cm/s) [8] (**Fig.** **5A**). By contrast, the permeability ratio of sodium over chloride (P_Na_/P_Cl_) between culture conditions (IPEC-J2/PS, P_Na_/P_Cl_ = 1.11±0.01, n = 13; IPEC-J2/FBS, P_Na_/P_Cl_ = 1.05±0.02, n = 11) was not significantly different and did not reach the degree of porcine jejunal epithelial cation selectivity (P_Na_/P_Cl_ = 1.44±0.07, n = 12, p<0.001) (**Fig. 5B**).

### PS elevates junctional and transport protein content

**Figure 6:**
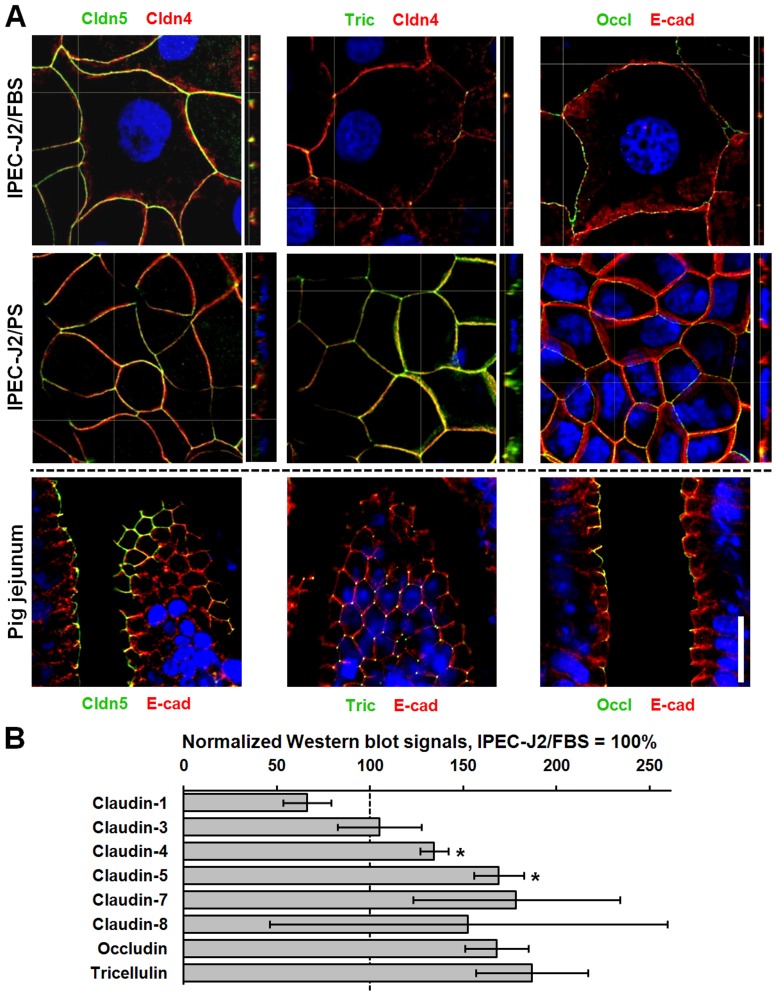
PS does not affect junctional protein localization within cell-cell contacts but controls tight junction protein quantity of IPEC-J2. (**A**) Confocal immunofluorescence images of IPEC-J2/FBS, IPEC-J2/PS, and cryosectioned pig jejunal mucosae. Cldn5, tric, and occl are presented in green, counterstain in red, as indicated. Nuclei are presented in blue (DAPI). The broken line indicates different counterstain between IPEC-J2 and pig jejunum. Scale bar: 20 µm. (**B**) Tight junction proteins of IPEC-J2/FBS and IPEC-J2/PS (n = 3 to 4 different cell passages) were analyzed by Western blotting and were subsequently densitometrically quantified. To allow for different cell architecture, values were normalized to E-cadherin. All signals of IPEC-J2/PS are given in relation to IPEC-J2/FBS values (100%). *, p<0.05.

The composition of the junctional protein pool and the presence of intestinal epithelial differentiation markers were studied by immunofluorescence staining. In pig jejunal epithelium, typical TJ proteins (claudin-1, -2, -3, -4, -5, -7, -8, -12, -15, tricellulin, occludin) which were selected on the basis of studies in mouse and rat small intestine [35]–[37], the TJ-associated protein Zonula occludens 1 (ZO-1), and the adherens junction protein E-cadherin (E-cad) were present and located within cell-cell contact complexes (**Figs.** **6A** **, S3, S4A**). Based on this, the expression patterns of differently cultured IPEC-J2 were screened. In IPEC-J2, claudin-1, -3, -4, -5, -7, and -8 could be detected within the TJ under both conditions (**Figs.** **6A, S** **3**), though claudin-7 localization within the TJ was patchy and weak (**Fig.** **S3**). Claudin-2, -12, and -15 signals were detected sporadically and faintly (**Fig.** **S4A**), but were not detectable on Western blots (data not shown) and regarding claudin-2 and -15 were also not present on mRNA level (**Fig. S4B**). Under both culture conditions tricellulin was expressed at tricellular junctions. However, bicellular localization was also observed, which occurred more frequently in IPEC-J2/PS than in IPEC-J2/FBS (**Fig.** **6A**). Occludin, ZO-1, and E-cad were located within the tight and adherens junction, respectively (**Figs.** **6A, S** **4A**). The quantification of proteins in Western blot analyses revealed a diverse pattern. Whereas expression of claudin-4 and -5 was increased (p<0.05) in IPEC-J2/PS, the content of other TJ proteins was not different from IPEC-J2/FBS (**Fig. 6B**).

**Figure 7:**
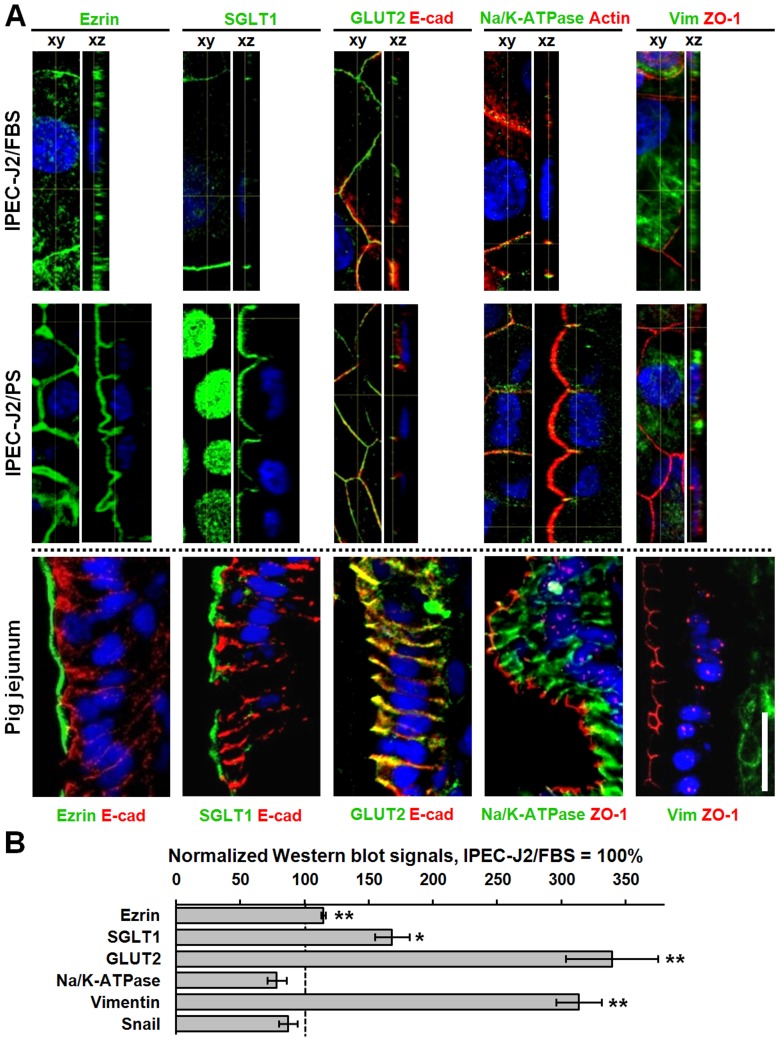
PS approximates porcine jejunal marker patterns of IPEC-J2 to that of jejunum. (**A**) Confocal immunofluorescence images of IPEC-J2/FBS, IPEC-J2/PS, and cryosectioned pig jejunal mucosae. Porcine jejunocyte marker proteins are presented in green, counterstain in red, as indicated. Nuclei are presented in blue (DAPI). In IPEC-J2, GLUT2 and Na/K-ATPase could be detected within the basolateral membrane, whereas ezrin and SGLT1 could be verified within the apical membrane of IPEC-J2/PS only. In addition, IPEC-J2 were positive for vimentin. The broken line indicates that counterstain choice differed between IPEC-J2 and pig jejunum. Scale bar: 20 µm. (**B**) Marker proteins of IPEC-J2/FBS and IPEC-J2/PS (n = 3 to 4 different cell passages) were analyzed by Western blotting and were subsequently densitometrically quantified. To allow for different cell architecture, values were normalized to E-cadherin, with the exception of vimentin, which was normalized to β-actin. All signals of IPEC-J2/PS are given in relation to IPEC-J2/FBS values (100%). *, p<0.05; **, p<0.01.

In terms of epithelial polarization markers, the presence of ezrin and SGLT1 within the porcine apical membrane as well as GLUT2 and Na/K-ATPase within the basolateral membrane could be confirmed (**Fig.** **7A**) and the absence of vimentin, a mesenchymal marker, within epithelial cells was verified (**Fig.** **7A**). Under both cell culture conditions, the expression of GLUT2 and Na/K-ATPase within the basolateral membrane could be proved (**Fig.** **7A**), whereas the expression of ezrin and SGLT1 within the apical membrane could be verified in IPEC-J2/PS only, since in IPEC-J2/FBS imaging apical and basal membranes spatially separately was difficult due to the low cell height (**Fig.** **7A**). However, against expectations, IPEC-J2 were positive for vimentin (**Fig.** **7A**). Protein quantities of ezrin and GLUT2 (p<0.01), SGLT1 (p<0.05), and the mesenchymal marker vimentin (p<0.01) were increased in IPEC-J2/PS, when compared to IPEC-J2/FBS (**Fig.** **7B**).

## Discussion

A cell culture model can serve as a powerful tool, provided it behaves like a functional and structural equivalent of the respective tissue. In case of the porcine small intestinal cell line IPEC-J2, cells should exhibit jejunal enterocyte features. While conventionally cultured IPEC-J2 were extremely large and flat and monolayers exhibited TER values at kΩ·cm^2^ level, jejunal enterocytes are small and columnar, and they exhibit TER values of only about 50 Ω·cm^2^. Considering that the jejunal lining is enlarged by villi and crypts, cell culture values were corrected using an enlargement factor of 10 which had been obtained from cross-sections of porcine jejunum. The resulting TER discrepancy between IPEC-J2 and jejunal tissue still amounts to one order of magnitude. In the current study we demonstrated that using a species-matched serum caused IPEC-J2 to develop an improved morphology accompanied by dramatically lower TER, resembling pig jejunocyte features.

### Relation between morphometry and TER

TER reflects two resistors in parallel, R^trans^ and R^para^. Assuming a constant R^para^ per unit TJ length and a constant number of ion channels per unit membrane, then cell layers consisting of small, tall cells will have lower TER values than cell layers consisting of large, flat cells. Microvilli and membrane invaginations which enhance the membrane area of the cells will further decrease TER [13]. Thus, it has been shown in a study which focused on a porcine colonic cell culture model [38], that cell monolayers possess adequate TER, which meet pig colonic tissue dimensions when corrected for the respective epithelial enlargement by crypts. Concomitantly, cells were found to exhibit appropriate cell dimensions, abundant and well-shaped microvilli as well as tight junctional and further intercellular contacts. A similar relationship between morphometry and TER has been observed in differently cultured human bronchial epithelial cells [39] as highly columnar shaped Calu-3 cells exhibit lower TER than cuboidal ones. In conventionally cultured IPEC-J2 monolayers, high TER values which do not correspond to the *ex vivo* counterpart are mainly a consequence of the enormous cell size. In addition, microvilli were small and sparse. In contrast, species-specific cultivation remodeled large and flat IPEC-J2 into smaller and taller ones with numerous long microvilli and this change in morphology was paralleled by a dramatic change in TER.

Previous ultrastructural studies demonstrated a correlation between the number of horizontal TJ strands and TER (or, more exactly, R^para^) [40], [41] even though other factors, such as TJ protein composition, evidently interferes with this correlation [42]. Thus, although in the current study total TJ length appeared to be the major determinant of TER, TJ ultrastructure may also have affected TER, as number of TJ strands and network depth were higher in IPEC-J2/FBS.

Besides TJ length and ultrastructure, the change in membrane area is likely to contribute to the drastic differences in TER between both culture regimes. These differences result from increased cell height, microvilli length and number, apical invaginations, and lateral infoldings in IPEC-J2/PS. The increase in membrane area can be directly deduced from the increase in epithelial capacitance observed during impedance measurements.

In IPEC-J2 of both kind, the ratio R^para^/R^trans^ is smaller than 1. Despite the high absolute resistances, this indicates a "leaky" epithelium [15] as it is typical for the jejunal lining.

Generally, the ion permeability of tight junctions is strongly augmented by the paracellular cation channels claudin-2 and claudin-15. Both claudins were present in porcine jejunum but were not detectable in IPEC-J2 of either kind. This may explain the lower cation selectivity of IPEC-J2 compared to that of jejunum.

Furthermore, forskolin-stimulated chloride secretion in IPEC-J2/PS was enhanced compared to IPEC-J2/FBS, indicating enhanced currents from the enlarged apical membrane area (dome-shaped, microvilli). Potential effects of higher channel density per unit membrane area were not investigated but may add to the observed current.

### Comparison of cell culture conditions inducing morphometry changes

Cells grown on membrane supports are commonly fed by medium from both the apical and the basolateral compartment. Due to poor IPEC morphology, Nossol and co-workers [32] suggested that the monolayer’s oxygen supply associated with ATP-dependent cellular biochemical and transport processes may be insufficient. In order to augment differentiation and functionality of IPEC monolayers, Nossol *et al*. performed ALI cultivation which optimized the cell height/diameter relation of IPEC-1 but did not improve these parameters in IPEC-J2. Inversely, TER of IPEC-1 did not show relevant changes two weeks after switching to ALI mode, whereas TER of IPEC-J2 declined by almost 50%, nevertheless retaining values at kΩ·cm^2^ level. The fact that morphometry and TER act contrarily to the TER/morphometry hypothesis postulated above indicates that TJ structure and/or membrane channel density must have changed greatly during ALI culture. As yet, the mechanisms responsible for changes in cell morphology under ALI conditions remain unclear.

Alternatively, it is not unlikely that morphological changes observed under ALI conditions and in the presence of PS are two sides of the same coin. Changes observed under ALI conditions may be caused by an accumulation of apically secreted substances within the very thin residual apical liquid layer. Thus, it can be hypothesized that IPEC-1 are able to synthesize components affecting cell morphology, whereas IPEC-J2 are not or only to a minor degree, so that IPEC-1 effectively accumulate signaling agents only when the apical extracellular volume is restricted. In porcine serum, whose proteome is not yet fully analyzed [43], all required signaling agents associated with differentiation/maturation presumably are included, so that IPEC-J2 morphometry and functionality was altered by replacing FBS with PS in culture media.

In all, FBS seems to be insufficient as medium supplement for IPEC-J2 culture although adequate cell morphometry and electrophysiology had initially been shown by Berschneider [4]. Porcine serum by contrast, apparently has the ability to compensate for a substance whose secretion by IPEC-J2 has been lost with increased number of passages. It needs to be elucidated, whether the saving serum agent is solely included in porcine serum and thus does not exist in bovine or goat sera of any age or whether its sequence differs between these species, leading to a loss of function of the potential bovine/goat analog in porcine cell culture.

### Differentiation and maturation

Once arisen from pluripotent stem cells located in the deep crypt epithelium, proliferative jejunocyte progenitor cells functionally and structurally start to differentiate during their migration along the crypt-villus axis. When the villous epithelium is reached, enterocyte differentiation is completed and jejunocyte maturation begins. The terms differentiation and maturation are often used interchangeably. However, differentiation describes quality changes, whereas maturation is the process of quantity changes in cell phenotype [44]. In order to judge the differentiation state of cultured epithelial cells, a variety of different markers could be used. Markers comprise cell type specific proteins which progressively complement the cell phenotype.

We tested protein expression of exemplarily chosen marker candidates, which are specific to polarized, barrier forming, and transport-active jejunal epithelial cells.

It was evident that all epithelial marker molecules tested, except claudin-2, -12, and -15, were qualitatively available under either growing condition. This suggests that IPEC-J2/FBS and IPEC-J2/PS display the same differentiation status. However, differences existed in terms of marker quantity. Compared to FBS condition, apical and basolateral marker proteins (ezrin, SGLT1, GLUT2) were significantly higher expressed, suggesting an advanced maturation state under PS culture.

Having shown that IPEC-J2 were differentiated, the mesenchymal marker vimentin, against expectations, was excessively expressed under both culture regimes. This phenomenon also appears e.g. in differentiated bovine primary culture jejunocytes, where it was attributed to a suppression of post transcriptional inhibition of the vimentin synthesis [45]. The appearance of vimentin may suggest an onset of epithelial-mesenchymal transition (EMT). EMT is triggered by several signaling pathways which induce the transcription factor snail. This, in turn, transcriptionally represses E-cadherin and TJ proteins [46] and in parallel increases vimentin expression [47]. Since snail expression was not elevated and junctional proteins were not down-regulated, a dedifferentiation of IPEC-J2 can be excluded. Incidentally, many established epithelial cell lines (MDCK-C7 [48], MDBK [49]) as well as epithelial cells *in vivo* [50] also express vimentin.

Together with respective protein qualities and quantities, the time course of TER reflects the differentiation/maturation state of either culture conditions. TER of IPEC-J2/FBS and IPEC-J2/PS reached highest values about one week post seeding which could indicate complete differentiation. Under FBS condition, TER values then remained at a more or less constant level, whereas under PS condition TER declined and reached lower plateau values arguing for a maturational process with leveled protein composition. In the absence of evidence to the contrary we can assume that the loss of internal maturational competence in IPEC-J2 is one disadvantage of non-immortalized continuous cell lines. In order to compensate such loss of function events, species-specific cultivation represents possibly the most effective proceeding.

## Conclusion

IPEC-J2 represent a unique tool for investigating porcine jejunal barrier function *ex vivo*. We describe an improved species-specifically cultured model, which exhibits morphology and barrier parameters close to the source tissue. This was urgently needed in order to replace rodent, tumor-derived, and non-physiologically behaving swine cell models in the field of pig small intestine and digestive research.

## Supporting Information

Figure S1 **PS does not change IPEC-J2 proliferation.** Metabolically active cells were photometrically quantified 4 and 72 h post seeding by applying the WST-1 assay. The absorbance at time point 4 h was set as 100%. n =  4 different cell passages; n.s., not significant.
(TIF)

Figure S2 **PS improves subcellular structures in IPEC-J2.** (**A**) Transmission electron microscopical (TEM) images (scale bar: 5 µm) of IPEC-J2/FBS and IPEC-J2/PS focusing on subcellular structures. M, mucopolysaccharides; MS, membrane support; MV, microvilli; N, nucleus. (**B**) More detailed TEM images representing tight junctional structures (scale bar: 1 µm). TJ, tight junction. (**C**) Paraffin sections of IPEC-J2/FBS and IPEC-J2/PS were stained for mucus by PAS reaction (scale bar: 20 µm). Neutral mucopolysaccharides which mainly existed in IPEC-J2/PS are depicted in pink and nuclei in dark blue.
(TIF)

Figure S3 **PS does not alter tight junctional protein localization in IPEC-J2.** Confocal immunofluorescence images of IPEC-J2/FBS, IPEC-J2/PS, and cryosectioned pig jejunal mucosae. Cldn1, -3, -7, and -8 are presented in green, counterstain in red, as indicated. Nuclei are presented in blue (DAPI). The broken line indicates that counterstain choice differed between IPEC-J2 and pig jejunum. Scale bar: 20 µm.
(TIF)

Figure S4 **Lacking claudins of IPEC-J2.** (**A**) Confocal immunofluorescence images of IPEC-J2/FBS, IPEC-J2/PS, and cryosectioned pig jejunal mucosae. Cldn2, -12 and -15 are presented in green, counterstain in red, as indicated. Nuclei are presented in blue (DAPI). The tested claudins were hardly detectable. The broken line indicates that counterstain choice differed between IPEC-J2 and pig jejunum. Scale bar: 20 µm. (**B**) mRNA isolated from IPEC-J2/FBS, IPEC-J2/PS, and pig jejunum was qualitatively analyzed by PCR. Cldn2, -12, and -15 mRNA bands (626 bp, 734 bp, 223 bp, respectively) of pig jejunum were used as a reference for IPEC-J2 in which only cldn12 could be verified. Negative controls are denoted by ‘/’.
(TIF)

Table S1 **Antibodies.**
(DOC)
