# Supplementary figures and images for: Improved Cell Line IPEC-J2, Characterized as a Model for Porcine Jejunal Epithelium
Source: PLoS One. 2013 Nov 15;8(11):e79643. doi: 10.1371/journal.pone.0079643 (PMC3829867; doi:10.1371/journal.pone.0079643)

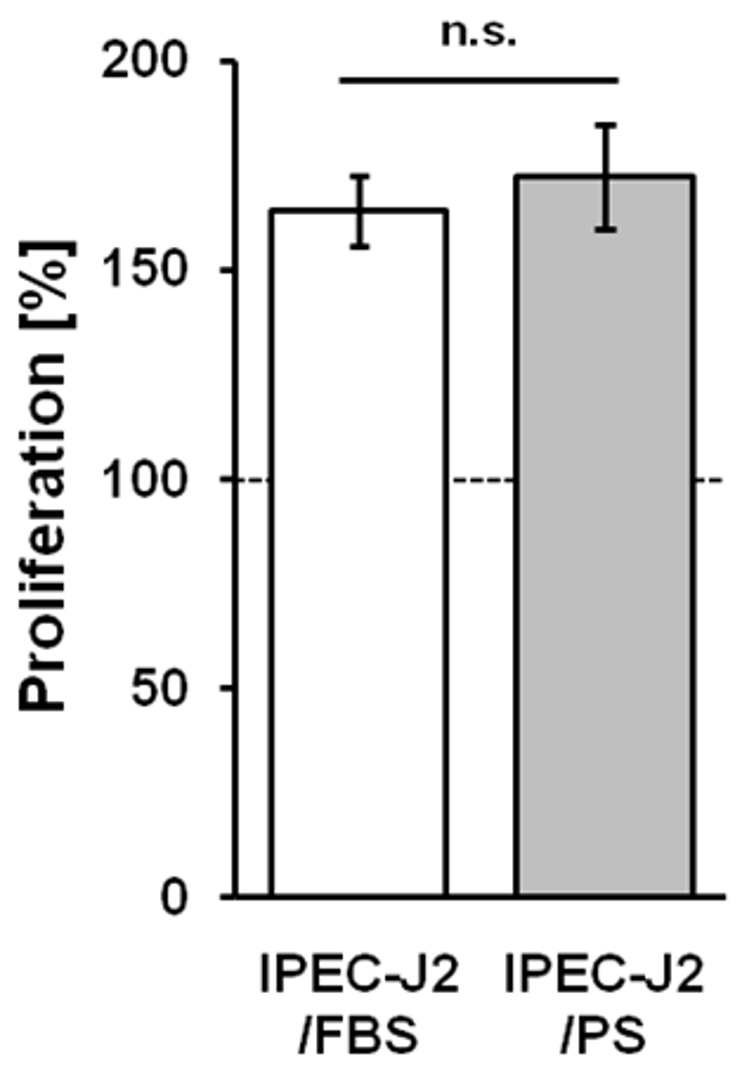

Supplement: Figure S1 — PS does not change IPEC-J2 proliferation. Metabolically active cells were photometrically quantified 4 and 72 h post seeding by applying the WST-1 assay. The absorbance at time point 4 h was set as 100%. n = 4 different cell passages; n.s., not significant. (TIF) [file pone.0079643.s001.tif]

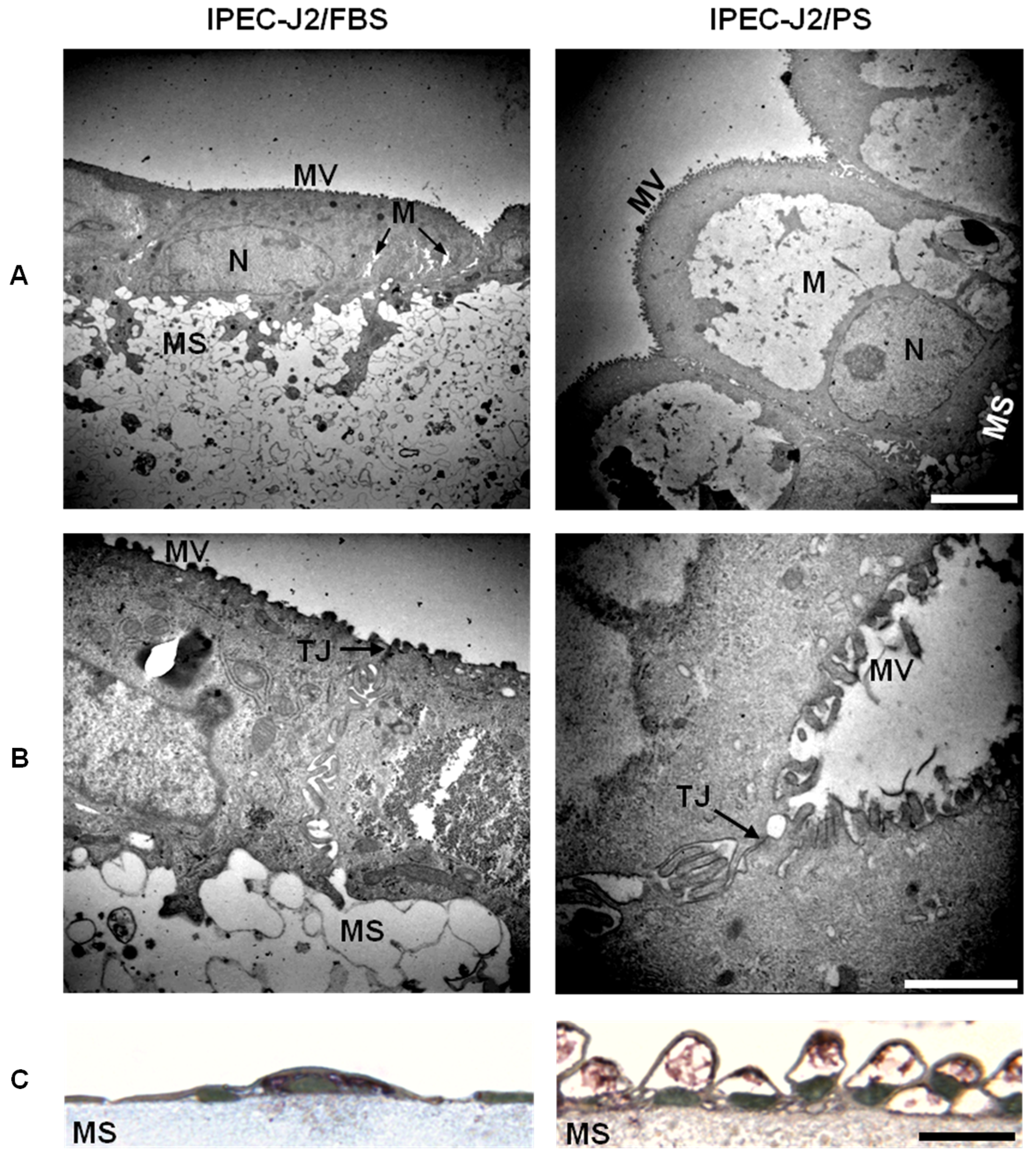

Supplement: Figure S2 — PS improves subcellular structures in IPEC-J2. (A) Transmission electron microscopical (TEM) images (scale bar: 5 µm) of IPEC-J2/FBS and IPEC-J2/PS focusing on subcellular structures. M, mucopolysaccharides; MS, membrane support; MV, microvilli; N, nucleus. (B) More detailed TEM images representing tight junctional structures (scale bar: 1 µm). TJ, tight junction. (C) Paraffin sections of IPEC-J2/FBS and IPEC-J2/PS were stained for mucus by PAS reaction (scale bar: 20 µm). Neutral mucopolysaccharides which mainly existed in IPEC-J2/PS are depicted in pink and nuclei in dark blue. (TIF) [file pone.0079643.s002.tif]

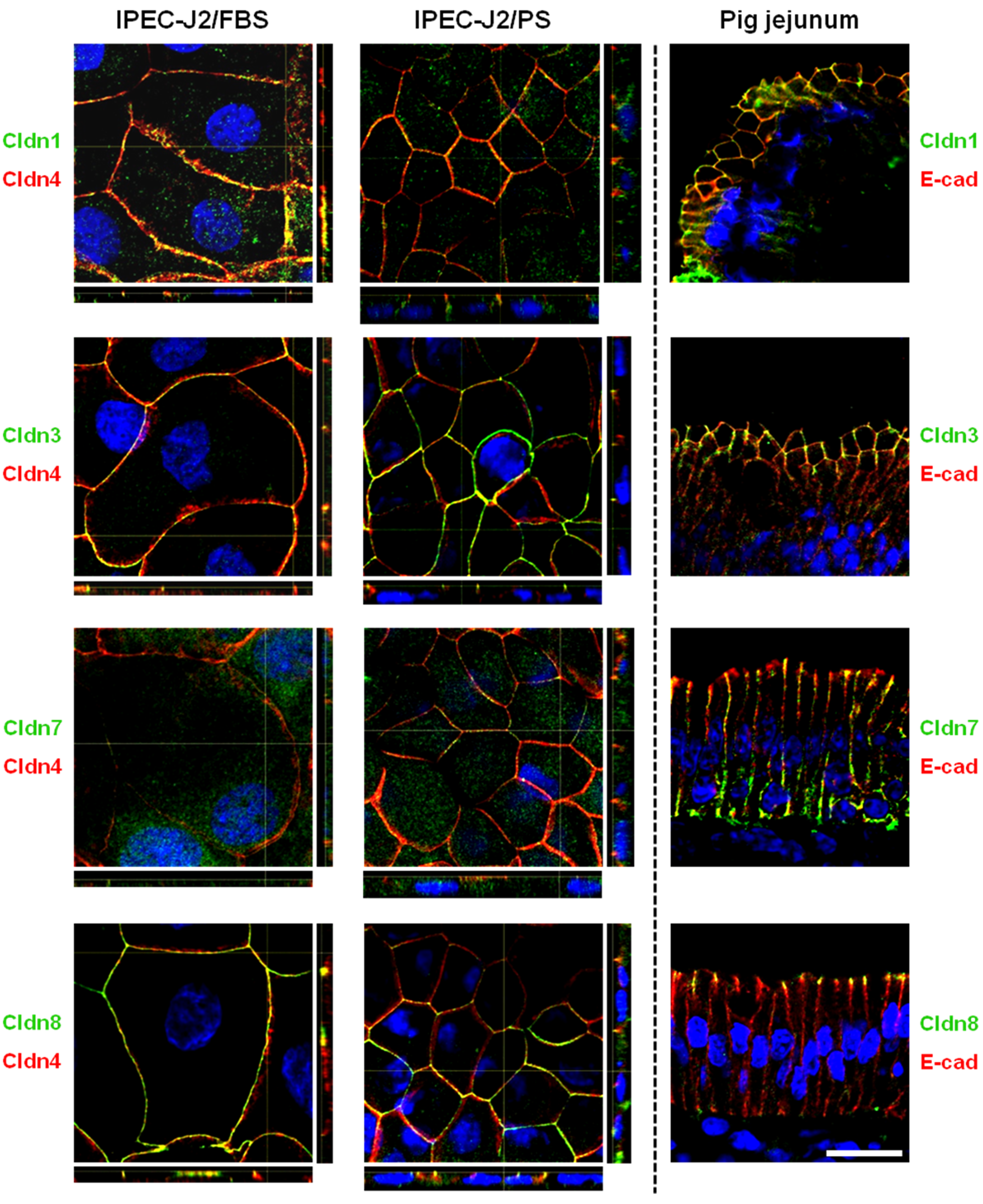

Supplement: Figure S3 — PS does not alter tight junctional protein localization in IPEC-J2. Confocal immunofluorescence images of IPEC-J2/FBS, IPEC-J2/PS, and cryosectioned pig jejunal mucosae. Cldn1, -3, -7, and -8 are presented in green, counterstain in red, as indicated. Nuclei are presented in blue (DAPI). The broken line indicates that counterstain choice differed between IPEC-J2 and pig jejunum. Scale bar: 20 µm. (TIF) [file pone.0079643.s003.tif]

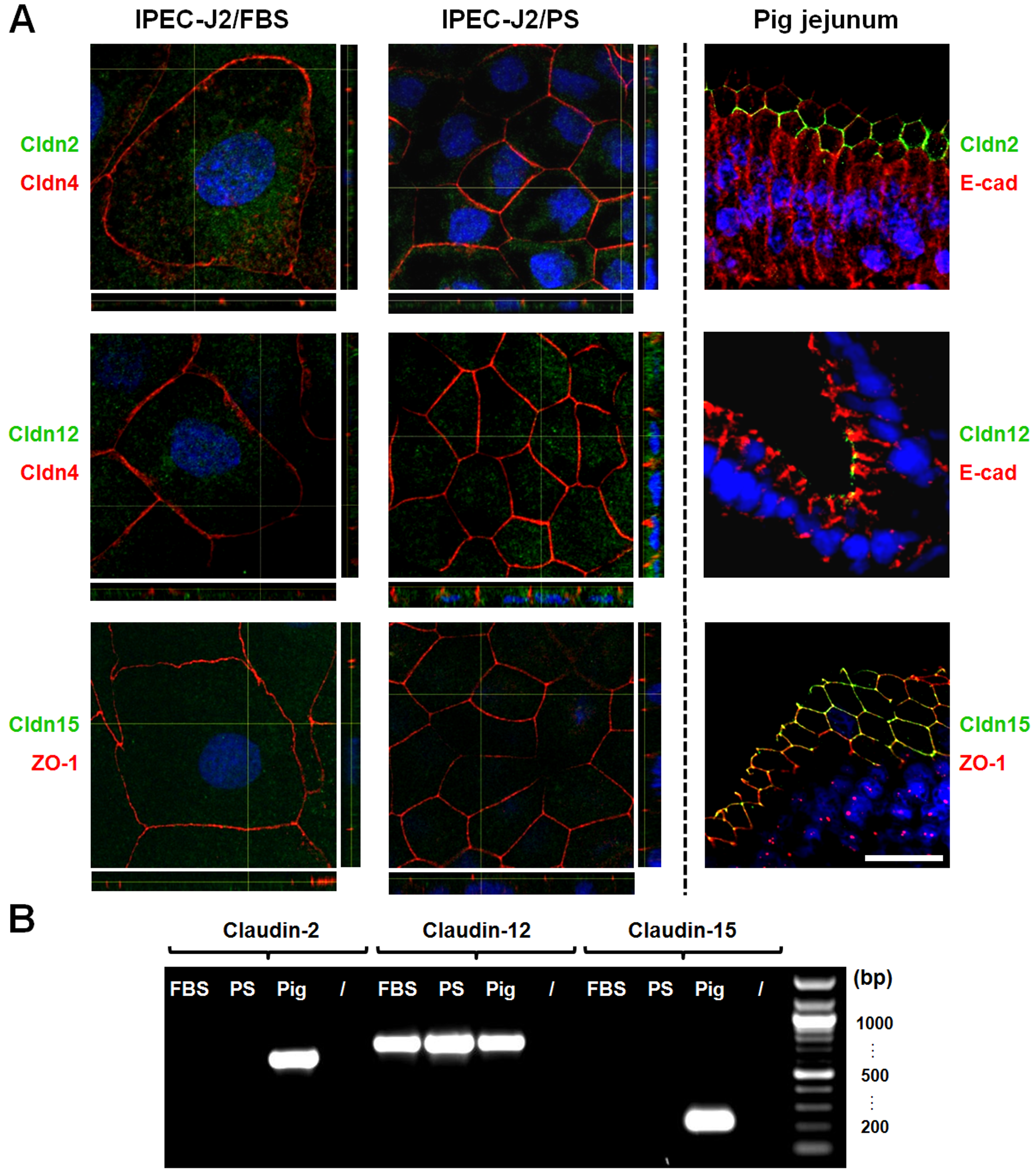

Supplement: Figure S4 — Lacking claudins of IPEC-J2. (A) Confocal immunofluorescence images of IPEC-J2/FBS, IPEC-J2/PS, and cryosectioned pig jejunal mucosae. Cldn2, -12 and -15 are presented in green, counterstain in red, as indicated. Nuclei are presented in blue (DAPI). The tested claudins were hardly detectable. The broken line indicates that counterstain choice differed between IPEC-J2 and pig jejunum. Scale bar: 20 µm. (B) mRNA isolated from IPEC-J2/FBS, IPEC-J2/PS, and pig jejunum was qualitatively analyzed by PCR. Cldn2, -12, and -15 mRNA bands (626 bp, 734 bp, 223 bp, respectively) of pig jejunum were used as a reference for IPEC-J2 in which only cldn12 could be verified. Negative controls are denoted by ‘/’. (TIF) [file pone.0079643.s004.tif]
