# Supplementary material for: Improved Cell Line IPEC-J2, Characterized as a Model for Porcine Jejunal Epithelium
Source: PLoS One. 2013 Nov 15;8(11):e79643. doi: 10.1371/journal.pone.0079643 (PMC3829867; doi:10.1371/journal.pone.0079643)
Supplement: Table S1 — Antibodies. (DOC) [file pone.0079643.s005.doc]

Table S1. Antibodies

| **Species** | **anti-** | **Target (+label)** | **Dilution (IF)** | **Dilution (WB)** | **Company** |
| --- | --- | --- | --- | --- | --- |
| *primary antibodies* | | | | | |
| rabbit | α- | Claudin-1/-2/-3/-7/-8/-12 | 1/250 | 1/1000 | Invitrogen, San Francisco, California |
| mouse | α- | Claudin-4 | 1/250 | 1/2000 | Invitrogen, San Francisco, California |
| rabbit | α- | Claudin-5 | 1/250 | - | Invitrogen, San Francisco, California |
| mouse | α- | Claudin-5 | - | 1/1000 | Invitrogen, San Francisco, California |
| mouse | α- | Claudin-15 | 1/250 | - | Invitrogen, San Francisco, California |
| rabbit | α- | Claudin-15 | - | 1/1000 | Aviva System Biology, San Diego, California |
| rabbit | α- | Occludin | 1/250 | 1/2000 | Invitrogen, San Francisco, California |
| rabbit | α- | Tricellulin | 1/200 | 1/2000 | Invitrogen, San Francisco, California |
| rabbit | α- | Zonula occludens 1 | 1/250 | - | Invitrogen, San Francisco, California |
| mouse | α- | E-cadherin | 1/250 | 1/3000 | Beckton Dickinson Transduction Laboratories™, France |
| mouse | α- | E-cadherin (AlexaFluor®647) | 1/1000 | - | Beckton Dickinson Pharmingen^TM^, France |
| mouse | α- | Vimentin | 1/250 | 1/1000 | Dako, Hamburg, Germany |
| rabbit | α- | Ezrin | 1/250 | 1/2000 | Cell Signaling Technology, Danvers, Massachusetts |
| rabbit | α- | SGLT1 | 1/250 | 1/1000 | Alpha Diagnostic International, San Antonio, Texas |
| rabbit | α- | GLUT2 | - | 1/2500 | Abbiotec, San Diego, California |
| goat | α- | GLUT2 | 1/250 | - | abcam, Cambridge, UK |
| mouse | α- | Na/K-ATPase, α1 subunit | 1/250 | 1/1000 | abcam, Cambridge, UK |
| goat | α- | Snail | - | 1/1000 | R&D Systems, Minneapolis, Minnesota |
| mouse | α- | β-Actin | - | 1/10000 | Sigma, Steinheim, Germany |
| *secondary antibodies* | | | | | |
| goat | α- | mouse (Cy2) | 1/500 | - | Jackson ImmunoResearch, Newmarket, UK |
| goat | α- | rabbit (AlexaFluor®594) | 1/500 | - | Invitrogen, San Francisco, California |
| rabbit | α- | goat (AlexaFluor®594) | 1/500 | - | Invitrogen, San Francisco, California |
| goat | α- | rabbit (DyLight^TM^488), F(ab')_2_ | 1/500 | - | Jackson ImmunoResearch, Newmarket, UK |
| goat | α- | mouse (peroxidase), F(ab')_2_ | - | 1/5000 | Jackson ImmunoResearch, Newmarket, UK |
| goat | α- | rabbit (peroxidase), F(ab')_2_ | - | 1/5000 | Jackson ImmunoResearch, Newmarket, UK |
| rabbit | α- | goat (peroxidase) | - | 1/5000 | Jackson ImmunoResearch, Newmarket, UK |
